# Comparative assessment of crystallographic and cryo‐EM models in the Protein Data Bank

**DOI:** 10.1002/2211-5463.70326

**Published:** 2026-08-26

**Authors:** Alexander Wlodawer, Pawel Rubach, Zbigniew Dauter, Wojciech Dec, Dariusz Brzezinski, Marta Kulik, Wladek Minor, Mariusz Jaskolski

**Affiliations:** ^1^ Laboratory of Cell Biology, Center for Cancer Research National Cancer Institute Bethesda MD USA; ^2^ Department of Molecular Physiology and Biological Physics University of Virginia Charlottesville VA USA; ^3^ Institute of Information Systems and Digital Economy Warsaw School of Economics Poland; ^4^ HKLResearch Charlottesville VA USA; ^5^ Department of Computational Biophysics and Bioinformatics Jagiellonian University Krakow Poland; ^6^ Doctoral School of Exact and Natural Sciences Jagiellonian University Krakow Poland; ^7^ Institute of Computing Science Poznan University of Technology Poland; ^8^ Faculty of Chemistry, Biological and Chemical Research Centre University of Warsaw Poland; ^9^ Institute of Bioorganic Chemistry Polish Academy of Sciences Poznan Poland; ^10^ Department of Crystallography, Faculty of Chemistry Adam Mickiewicz University Poznan Poland

**Keywords:** cryo‐EM, cryo‐EM validation, crystallography, Electron Microscopy Data Bank, Protein Data Bank

## Abstract

With cryogenic electron microscopy (cryo‐EM) on track to surpass X‐ray crystallography as the preferred method for determining macromolecular structures, it is important to evaluate and compare the quality of structure models obtained by these methods. This allows us to assess whether the rapidly growing numbers (quantity) correlate with quality and to identify areas in which each method excels or falls short. Selected quality‐related parameters were compared for 97 200 crystal structures and 30 139 cryo‐EM structures released by the Protein Data Bank (PDB) between 2015 and 2025. Comparison of geometric and stereochemical parameters indicated that, despite significant differences in the resolution of the experimental data, these values were, in the vast majority of cases, close to the expected targets. Nevertheless, we found that crystal structures tend to exhibit more Ramachandran and rotamer outliers than cryo‐EM structures, although they unexpectedly have lower clashscore values. Separately, we compared the quality of 612 crystal and 1817 cryo‐EM structures in the PDB representing complete ribosomes or their subunits. For this subset of very large, well‐defined macromolecules, we found that the quality of many cryo‐EM models is higher than that of their crystal counterparts, and that the best cryo‐EM structures were also determined at higher resolution. Overall, we conclude that the availability of both techniques has clearly resulted in major advances during the last decade and bodes very well for the future.

## Graphical abstract

Raw data obtained by X‐ray crystallography or cryo‐EM result in experimental maps, ultimately fitted by atomic models. Although the physical principles are different, the final results can be viewed, compared, and evaluated in the same way.

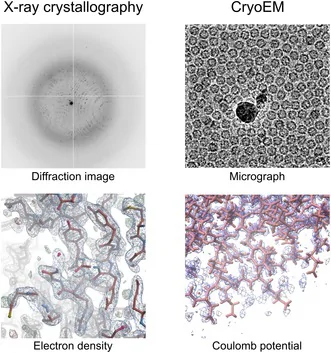

**Abbreviations**
ADP: atomic displacement parameter
AI: artificial intelligence
EMDB: Electron Microscopy Data Bank
FSC: Fourier Shell Correlation
PDB: Protein Data Bank
RCSB: Research Collaboratory for Structural Bioinformatics
RMSD: root‐mean‐square deviation
RSRZ: real‐space *R*‐factor Z‐score

The last decade has witnessed major advances in experimental techniques for determining medium‐ to atomic‐resolution structures of biological macromolecules. Whereas X‐ray crystallography was the source of 88.8% of all deposits made since the creation of the Protein Data Bank (PDB) in 1971 [1] until the end of 2014, single‐particle imaging by cryogenic electron microscopy (cryo‐EM) turned recently into the first‐choice method, particularly for large macromolecules and their assemblies, as well as for transient complexes. The number of crystal structures deposited in the PDB each year in the last decade has been almost constant at about 8000, while the number of cryo‐EM deposits has grown very rapidly and appears poised to surpass the number of annual crystallographic depositions in the near future (Fig. 1).

**Fig. 1:**
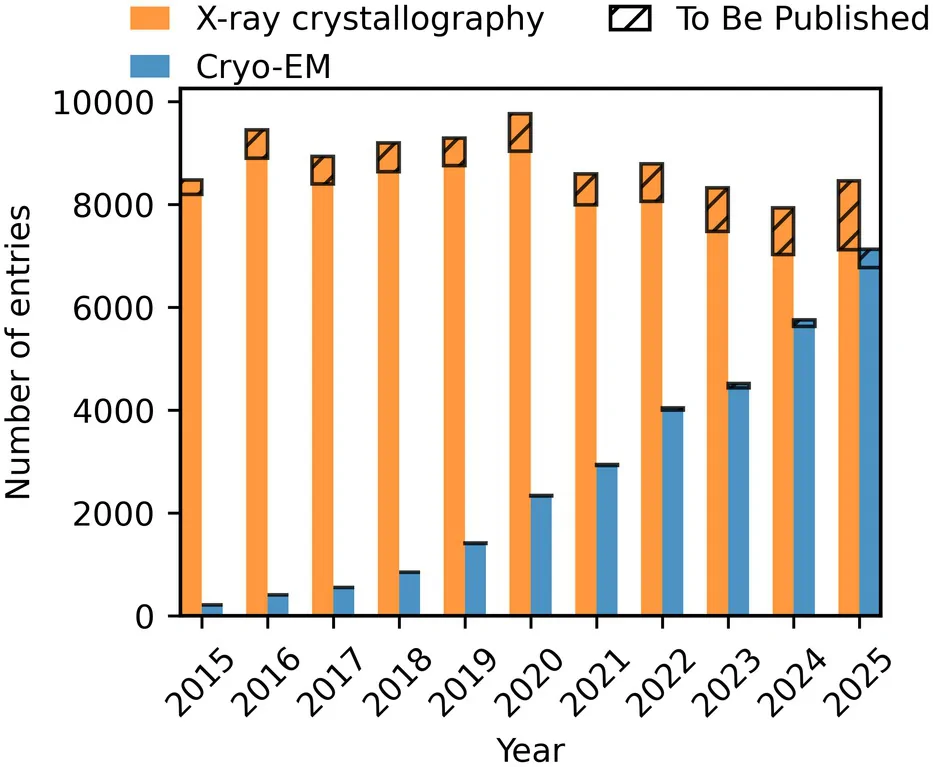
Number of yearly Protein Data Bank (PDB) deposits of structures determined by X‐ray crystallography and by cryo‐EM released between 2015 and 2025. Hatched parts of the bars represent deposits with no reference in the PDB to a publication.

However, the two techniques are often applied to different classes of macromolecules, achieve different levels of accuracy of the resulting atomic coordinates, and are likely to suffer from different types of problems, errors, and blunders. A summary of the advantages and disadvantages of different structure determination methods in obtaining quality information, especially for ligand complexes, has been published previously [2]. Yet maintaining the highest quality of the data in the PDB, regardless of the experimental method, is of critical importance for a variety of reasons. For instance, biologists often use individual structures deposited in the PDB to guide modification of protein or nucleic acid function, or to search for novel drugs. On the other hand, the whole contents of the PDB are used for data‐mining campaigns and, even more importantly, have been used as the main training set for artificial‐intelligence‐based structure prediction software, such as AlphaFold2 [3], RoseTTAFold [4], and other similar programs. With many users of the PDB data not being well‐versed in the intricacies of the methodologies (or even not being human at all), it is important to assess the apparent quality of individual structures, as well as to make global comparisons of quality indicators across structures determined by different methods. In this work, we are concerned with the two major techniques for structure determination: macromolecular crystallography and single‐particle imaging using high‐resolution cryo‐EM.

Assessment of the quality of macromolecular structures involves several aspects; accordingly, a number of different quality‐related indicators can be found in the validation reports prepared for each deposit by the PDB, as well as in the mmCIF (and legacy PDB) files that contain the atomic coordinates. Some indicators report the plausibility of the stereochemical (or geometric) aspects of the macromolecular models. These indicators are the same for structures determined by crystallography and cryo‐EM and, for proteins, report the presence of Ramachandran outliers, the number of close contacts between atoms (per 1000 non‐H atoms) leading to forbidden clashes (clashscore), and nonrotameric conformations of the side chains (sidechain outliers). The values of these parameters are reported by the RCSB PDB for the vast majority of deposits in a prominent graphical and numerical form at the top of the web page for each entry, as well as in the validation reports generated and available for each deposit. It is somewhat surprising that nearly 40% of all cryo‐EM deposits lack information on refinement [2], which may indicate either that the models were not refined, refined by several programs, or that the depositors did not consider this information important enough to report.

Some of the newer PDB entries that contain RNA chains now also include a slider referred to as the ‘RNA backbone suite’ metric [5], with values extending from 0 (worst) to 1 (best). This metric evaluates the likelihood of the presence of known conformers linking two consecutive nucleotides. The outliers are marked in the validation report for individual nucleotides, and the average value of the ‘suiteness’ parameter is shown in the slider. A newer quality assessment system for nucleic acid structures was recently proposed [6], but has not yet been implemented by the PDB as of the time of this writing.

The other aspect of structure quality concerns the agreement of the deposited model with the experimental data, and it is not necessarily the same for crystal and cryo‐EM deposits. A common feature of such data is its resolution, although it is not defined in the same way for the two techniques. In crystallography, resolution corresponds to the *d*_min_ limit calculated from Bragg's Law and corresponding to the highest scattering angle where diffraction is still detectable, according to criteria such as <I/σ> (average of reflection intensity divided by its standard deviation), CC_1/2_ (correlation coefficients between two half‐sets of the experimental intensity data), or CC* [estimate of the correlation between the experimental intensities and the true (but unknown) intensities] reaching a predetermined limit (although the values of such limits are still subject of discussion) [7]. The usual way of estimating overall resolution of cryo‐EM data is by analyzing the value of FSC143, a point where the correlation coefficient between two independently calculated half‐maps reaches 0.143 [8]. It was previously shown that maps calculated by crystallography and cryo‐EM at the same apparent resolution may differ in their appearance [9].

It is important to remember that the maps (which are the primary results of both experiments) obtained by the two methods are not equivalent. X‐ray crystallography produces electron density maps, showing the distribution of electrons in the molecules and their crystals. Cryo‐EM maps (further abbreviated as EM maps), on the other hand, display the electrostatic potential in the molecules, to which both the electrons and atomic nuclei contribute. A stark illustration of this difference is the impossibility of locating protons (H^+^) in electron density maps and their visibility in EM maps. All EM maps are deposited in the Electron Microscopy Data Bank (EMDB), whereas a single map corresponding to the final cryo‐EM structure is deposited in the PDB, together with the relevant atomic coordinates. Subsequently, the agreement between the model and the map, perhaps the most important indicator of structure quality, is reported differently for crystal and cryo‐EM structures.

In this work, we compare the overall quality indicators of all crystal and cryo‐EM structures released by the PDB between January 1, 2015, and December 31, 2025. We selected that range of dates since it corresponds to the revolutionary developments in cryo‐EM that resulted from the introduction of direct electron counting detectors, rapid sample vitrification in liquid ethane, and modern software [10, 11]. We also provide a separate comparison of crystal and cryo‐EM structure quality for ribosomes, a specific group of large macromolecules containing both proteins and RNA, that illustrates the current shift of preferences in using cryo‐EM as a principal method of macromolecular structure determination.

## Materials and methods

### Selection of data for analysis

Data used for the comparison of quality parameters of models determined by X‐ray crystallography and cryo‐EM consisted of all PDB deposits that included at least one protein chain, released between January 1, 2015, and December 31, 2025 [97 200 crystal structures (Table S1)] and 30 139 structures determined by electron microscopy, almost all by cryo‐EM (Table S2). All entries labeled ‘Group deposition’ were excluded from the analysis.

For a separate comparison of only ribosome structures, we selected deposits identified in the header as ‘RIBOSOME’ and having a molecular mass in excess of 600 kDa. Such deposits included complete ribosomes from different organisms and organelles or at least their small or large subunits, but excluded isolated ribosomal proteins. For these datasets, we did not impose any limits on when the structures were released by the PDB. We found 612 crystal structures (Table S3) and 1817 cryo‐EM structures (Table S4) satisfying these selection criteria. It is possible that there are still other ribosome structures present in the PDB that are not marked as such; an example is entry 5njt, marked as ‘TRANSLATION’ and thus not selected by the criteria listed above.

### Structure and ligand quality metrics

For crystal structures, it is possible to calculate the *R* factors that describe the discrepancy between the measured structure factor amplitudes and their model‐derived values. In particular, *R*_free_ [12] is based on a subset of reflections that were not used in structure refinement and are thus free of bias introduced during the refinement. This parameter is useful for assessing global agreement between the model and the diffraction data, but it will not pinpoint specific places where the model does not agree with the electron density map. For this purpose, a parameter called RSR (real‐space *R*‐factor) is used. It evaluates the agreement of individual residues of the model (represented by their calculated electron density) with the experimental electron density map. For convenient presentation, the RSR values are normalized against the expected values for a specific residue type and resolution bin, and their *Z*‐score is calculated as RSRZ. For crystal structures, the RSRZ parameter is addressed by giving the number of outliers, defined as the percentage of values deviating from the average by more than 2σ [13]. Another metric describing per‐residue agreement between the observed and calculated electron density maps is the real‐space correlation coefficient, RSCC. Shao et al. [14] provided such computations for over 100 million cases of standard amino acids and nucleotides found in the PDB.

The single‐parameter quality metric PQ1 [15, 16] was introduced to broadly assess the quality of the entire set of crystal structures. PQ1 provides a fractional score reflecting the relative structural quality of a given entry compared to the entire PDB. This score integrates *R*_free_, normalized real‐space *R*‐factor outliers (RSRZ), Ramachandran outliers, rotamer outliers, and clashscore. PQ1 values range from 0 to 1, representing, respectively, the PDB's lowest and highest quality structures. For this study, PQ1 calculations were performed against all PDB entries determined by X‐ray crystallography and deposited between 2015 and 2025, inclusive (Table S1).

A set of metrics typically applied to cryo‐EM structures includes correlation between the experimental EM map and an analogous map calculated on the basis of model coordinates. Different definitions and algorithms for calculating such correlations are described in detail by Afonine et al. [17]. CC_map_ gauges similarity between the entire experimental EM map and a calculated density map generated from an atomic model, whereas CC_mask_ limits the comparison to the area masked by the coordinates. CC_map_ (and CC_mask_) correlation coefficients are present in the validation reports for a majority of cryo‐EM entries in the PDB/EMDB. Another quality parameter reported for more recent cryo‐EM PDB deposits is *Q*‐score, a metric that measures the local resolvability of individual atoms or residues within a cryo‐EM map [18]. It ranges from −1 to 1 and has been shown to be strongly correlated with map resolution [19], in some cases becoming negative when the map and the coordinates are anticorrelated [2]. Following a community recommendation [20], *Q*‐scores are now included in the official validation reports for PDB/EMDB models. The quality parameters for cryo‐EM structures deposited between 2015 and 2025 are provided in Table S2.

Each Supplementary Table contains three pairs of columns: Clashscore and VR Clashscore; Rotamer outliers and VR Rotamer outliers; and Ramachandran outliers and VR Ramachandran outliers. Columns prefixed with ‘VR’ contain data extracted from the XML versions of validation reports published by the PDB, whereas the corresponding columns without a prefix were computed by us using the MolProbity tool distributed with CCP4 (version 9.0.014). The Molprobity computed values were used in our analysis. This approach ensures greater comparability across structures than relying directly on the validation report values. Validation reports are typically generated during the structure deposition process and are very infrequently updated thereafter, making it difficult to determine the exact version of MolProbity used to calculate the reported geometrical parameters. As our earlier analysis has shown, [2, 21] different versions of MolProbity can yield slightly different results. Although the PDB provides the version of CCP4 used during processing, this information does not necessarily identify the specific MolProbity version employed. To address these inconsistencies, we recalculated all values using a uniform MolProbity version for all structures. Additionally, we identified a minor issue in the validation report data: in 44 PDB deposits the reported clashscore value is −1.

## Results

### Distribution of data resolution and of the size of crystal and cryo‐EM structures

**Fig. 2:**
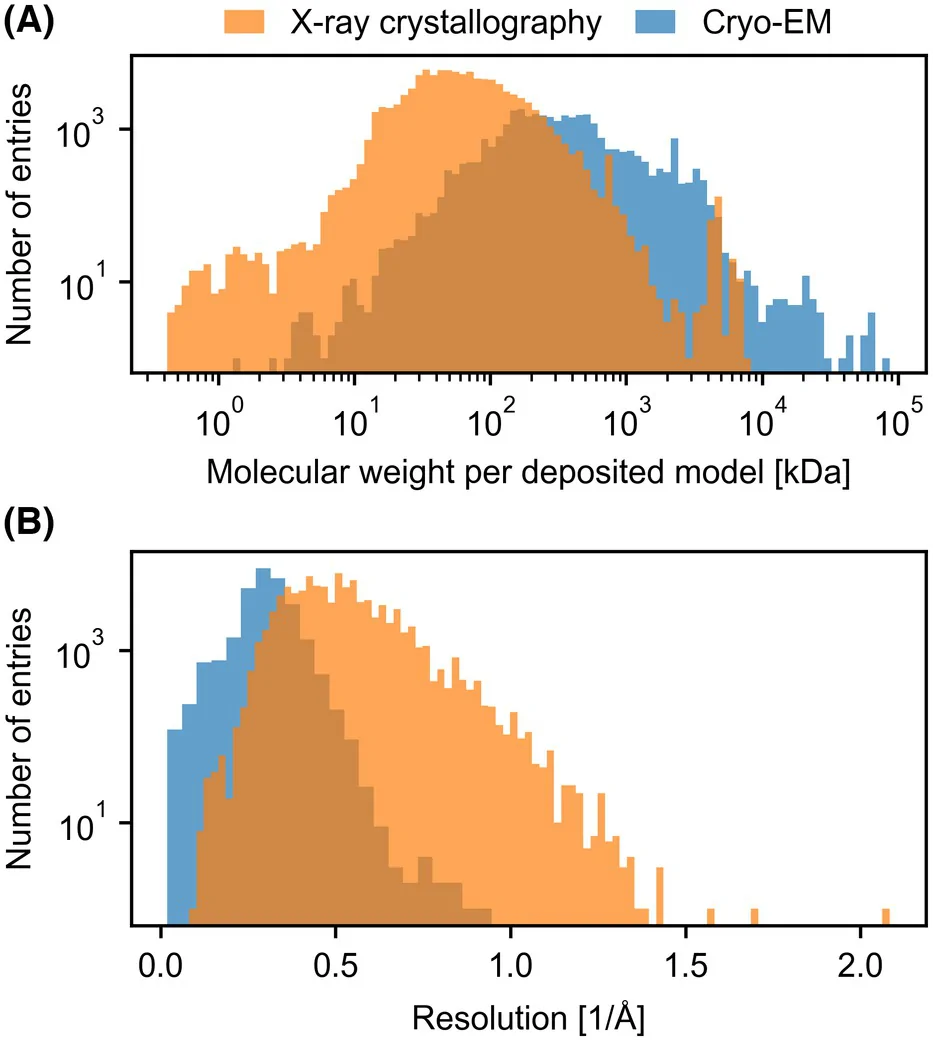
A comparison of the sizes of macromolecules in crystal and cryo‐EM structures deposited in the PDB from 2015 to 2025 (97 200 and 30 139, respectively), and of the resolution of the corresponding experimental data. (A) A histogram of the distribution of molecular weight for X‐ray crystallographic and cryo‐EM models. (B) A histogram of the distribution of the claimed resolution for X‐ray crystallographic and cryo‐EM structures. Seven cryo‐EM structures with missing values for resolution are not included. The y‐axes of both panels and the *x*‐axis of panel A are presented on a logarithmic scale. Resolution in panel B is expressed as 1/*d*_min_.

The sizes of structures deposited in the PDB can be evaluated on the basis of two largely redundant parameters listed in the deposits. This information can be gleaned from either the number of nonhydrogen atoms present in the deposited models or the molecular weight of the content of the asymmetric unit of the crystals or of the total cryo‐EM model. For cryo‐EM structures with internal symmetry, the size may represent the entire biological unit or just a part of it, whereas in crystallography, a symmetric assembly occupying a special position in the unit cell will be represented by its part (such as a protomer) only, or conversely, there may be multiple copies of the macromolecule in the asymmetric unit. Entry size distribution, showing the number of entries within selected size limits for deposits released between 2015 and 2025, is illustrated in Fig. 2A. The range of molecular weight of the models obtained by cryo‐EM ranges from 1.43 kDa (9fck) to 86 760 kDa (9fqr). The molecular weight of models determined by X‐ray crystallography ranges from 0.42 kDa (5zck) to 7655 kDa (5vyc). The distribution indicates very clearly that crystal structures still predominate for smaller macromolecules, whereas the largest structures are accessible only to investigations by cryo‐EM.

Distribution of the resolution reported for crystal and cryo‐EM structures in the PDB during the period 2015–2025 is shown in Fig. 2B and is quite different for the two methods. Resolution of crystal structures ranges from 0.48 Å (5d8v) to 10 Å (5l5g), whereas the range of cryo‐EM structures is from 1.09 Å (8rqb) to 53 Å (6u3g). The median value of resolution of crystal structures is 2.0 Å, whereas the median value for cryo‐EM structures is 3.3 Å. Seven cryo‐EM deposits do not list resolution at all. Five of them are tomography‐derived models fitted without further refinement (3jc8, 3jc9, 3jcb, 3jcc, 8gy6), while the other two deposits (7mbu and 7mbt) are cryo‐EM structures and the missing resolution may be a result of deposition errors.

### Comparison of geometric and stereochemical parameters

The indicators of the geometric and stereochemical parameters of both the crystal and cryo‐EM models span a large range of values. The clashscore values of the crystal structures range from the ideal value of zero (685 deposits) to the rather unlikely 169.6 (5xa0), but are not listed in 3998 deposits. For cryo‐EM models, clashscores range from zero (216 entries) to over 200 (11 entries), with an extreme case of clashscore 348.9 (6c06). It should be noted that for crystallographic models clashscore includes both intramolecular as well as intermolecular contacts, mostly involving crystal packing. For cryo‐EM deposits, the latter are not applicable. This aspect should be taken into account when comparing the clashscore metric for crystallographic and cryo‐EM models. Zero rotamer outliers are reported for 8208 cryo‐EM models, whereas 547 models have over 10% outliers. Surprisingly, five cryo‐EM models (8zxl, 9vnk, 8z94, 9vnm, and 7cr4) with no rotamer outliers have clashscore exceeding 100, indicating that each of these different indicators may not be sufficient by itself to judge structure quality. For crystal structures, zero rotamer outliers are reported for 16 083 deposits, and 2071 structures are listed with more than 10% outliers.

**Fig. 3:**
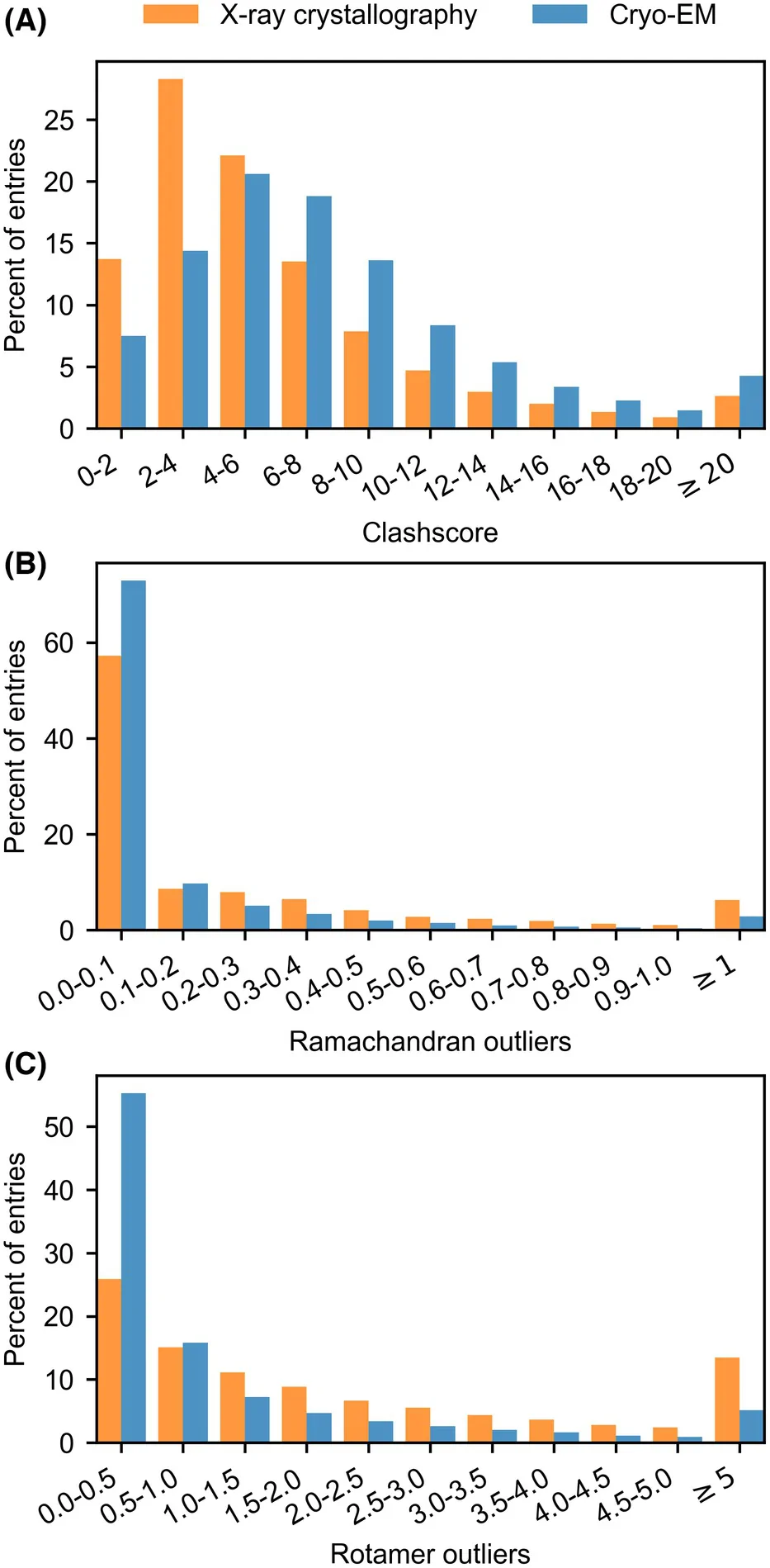
Histograms of the distribution of clashscore (A), Ramachandran (B) and rotamer (C) outliers (%), for X‐ray crystal and cryo‐EM structures determined within the resolution range 1.8–4.0 Å. Structures determined by X‐ray crystallography and cryo‐EM with missing values (3119 and 40, respectively) are not shown.

A direct comparison of the geometric parameters of crystal and cryo‐EM structures is meaningful only within resolution limits that exclude ultrahigh resolution, which is accessible only to crystallography (see below) and the very low resolution found for many cryo‐EM structures, but rarely for crystal structures (Fig. 2B). We realize that setting such limits is quite arbitrary, but for the purpose of the comparisons presented here, we set the limits between 1.8 and 4 Å. Thus, we directly compared 67 027 crystal structures with 25 205 cryo‐EM structures within this resolution range. Compared indicators describing the geometrical and stereochemical quality of both X‐ray crystal and cryo‐EM models include Ramachandran and rotamer outliers, as well as clashscore (Fig. 3).

Surprisingly, although clashscore values for crystal structures include crystal contacts that are absent in cryo‐EM structures, the percentage of crystal structures with clashscore below 5 (54%) is significantly higher than for cryo‐EM structures (32%). The opposite trend is seen in all larger clashscore ranges, indicating that this particular metric is worse in cryo‐EM than in crystal structures. A vast majority of both crystal and cryo‐EM structures (93% and 97%, respectively) show none or at most 1% of Ramachandran outliers, with the count of outliers in crystal structures exceeding that in cryo‐EM structures in almost all other ranges. A similar situation is encountered for rotamer outliers, where a significantly larger fraction of cryo‐EM structures (83%) is observed with fewer than 2% outliers than for crystal structures (61%), with the same trend continuing in other ranges.

### Cryo‐EM structures at near‐atomic resolution

Seven cryo‐EM structures were determined during the last 5 years at a resolution reaching or approaching the atomic level (here somewhat arbitrarily set at 1.3 Å). Six of these structures represent the heavy chain of mouse or human apoferritin (8rqb, 7a6a, 8j5a, 7a4m, 6z6u, and 7rrp), whereas the structure 9zc2 represents the DNA protection protein during starvation (DPS), related to ferritin. These proteins are all highly symmetric, with 24 apoferritin protomers forming a cluster with point group symmetry 432 (*O*) and 12 DPS protomers arranged according to point group 23 (*T*). The symmetric nature of these proteins was utilized directly during structure determination and was crucial for calculating EM maps at such high resolution. Geometric and stereochemical parameters of these structures, as shown in the original validation reports, are also excellent, with no Ramachandran outliers, side chain outliers at 0–2.6%, and clashscore of 0–3.0. No *Q*‐score values are available for models 8rqb, 7a6a, 8j5a, and 7a4m, whereas the other three models have high *Q*‐scores ranging from 0.908 (7rrp) to 0.934 (9zc2). CC_mask_ parameters range from the low of 0.571 for 7a4m to the high of 0.854 for 9zc2.

Although as many as 5527 crystal structures deposited between 2015 and 2025 were determined at a resolution of 1.3 Å or better, none of them were of either apoferritin or DPS. The only crystal structure of apoferritin determined at a similarly high resolution (1.15 Å) represents a mutant (E53,56,57,60Q, and R59M) of the light chain of horse spleen ferritin (2v2p). However, that crystal structure, deposited about 15 years ago [22], is by current standards of mediocre quality (PQ1 0.48, *R*_free_ 0.224, clashscore 10, sidechain outliers 0.7%, RSRZ outliers 4.1%), despite the fact that the crystal asymmetric unit in the cubic space group *F*432 contained only a single protein chain and the full symmetry of the molecule was expressed by crystal symmetry. Two DPS crystal structures determined in the native form or soaked with iron (3ak8 and 3ak9; [23]) were of higher quality, although still not matching the quality of the more recently solved cryo‐EM counterparts. They were determined at the resolution of 1.25 and 1.3 Å, respectively, and characterized by *R*_free_ of 0.204 and 0.181.

### Comparison of the quality of crystal and cryo‐EM structures of smaller proteins

Whereas a majority of cryo‐EM structures were determined for comparatively large macromolecules or macromolecular complexes (Fig. 2A), there have been several tests utilizing this technique for investigation of smaller objects, with molecular weight on the order of 50–100 kDa. The tests were performed for proteins such as human methemoglobin (6nbc and 6nbd; [24]), horse liver dehydrogenase (6nbb; [24]), malate synthase G (7yqm; [25]), and streptavidin (6j6j and 6j6k; [26]), among others. All these cryo‐EM structures were determined at a resolution ranging from 2.9 to 3.3 Å, very significantly lower than the resolution of the corresponding crystal structures that often reached atomic resolution. However, later cryo‐EM investigations (some of them still unpublished) of the structure of streptavidin extended the resolution to as high as 1.7 Å, meriting a more detailed comparison.

Streptavidin is a 222 (*D*_2_) homotetramer with a total molecular weight of ~ 52 kDa. Cryo‐EM structures of the protein only (6j6k) and of its complex with biotin (6j6j) were determined utilizing averaging according to the *D*_2_ symmetry at a resolution of 3.3 Å and 3.2 Å, respectively. These structures are characterized by respective *Q*‐scores of 0.497 and 0.517, and clashscores of 5.53 and 6.72. Rotamer outliers of 1.16% and Ramachandran outliers of 2.6% were reported for the former structure, whereas no rotamer or Ramachandran outliers were reported for the latter one. Resolution of the subsequently determined cryo‐EM structure 8hrm [27] was significantly higher (2.56 Å), but its *Q*‐score of 0.518, clashscore of 18.48, rotamer outliers of 5.83%, and Ramachandran outliers of 0.85% indicate a comparatively low quality. Structure 8gvk, determined at 2.2 Å resolution [28], was of slightly higher quality (*Q*‐score 0.654, clashscore 5.53, rotamer outliers 2.62%, Ramachandran outliers 2.56%). Three unpublished high‐resolution structures (7dy0, 7efd, and 7efc) represent different selection of frames from a single dataset and were determined at the respective resolution of 1.93, 1.77, and 1.70 Å. Only a single streptavidin protomer was deposited, with reconstruction of the tetramer requiring application of the *D*_2_ symmetry. Structure 7efc was characterized by *Q*‐score of 0.708, clashscore 1.61, rotamer outliers 2.27%, and 0.85% Ramachandran outliers.

Over 260 crystal structures of streptavidin have been deposited since 1992. Their resolution ranges from the low of 2.9 Å (1rxh) to the high of 0.85 Å (2f01; [29]). The ‘best’ crystal structure according to the PQ1 indicator of 0.89 was determined by native SAD at 1.55 Å resolution (6m9b; [30]). It is characterized by *R*_free_ 0.183, clashscore 1.68, no rotamer or Ramachandran outliers. Comparable parameters for the highest‐resolution structure 2f01 are PQ1 0.60, *R*_free_ 0.174, clashscore 7.91, rotamer outliers 2.8%, and no Ramachandran outliers.

**Fig. 4:**
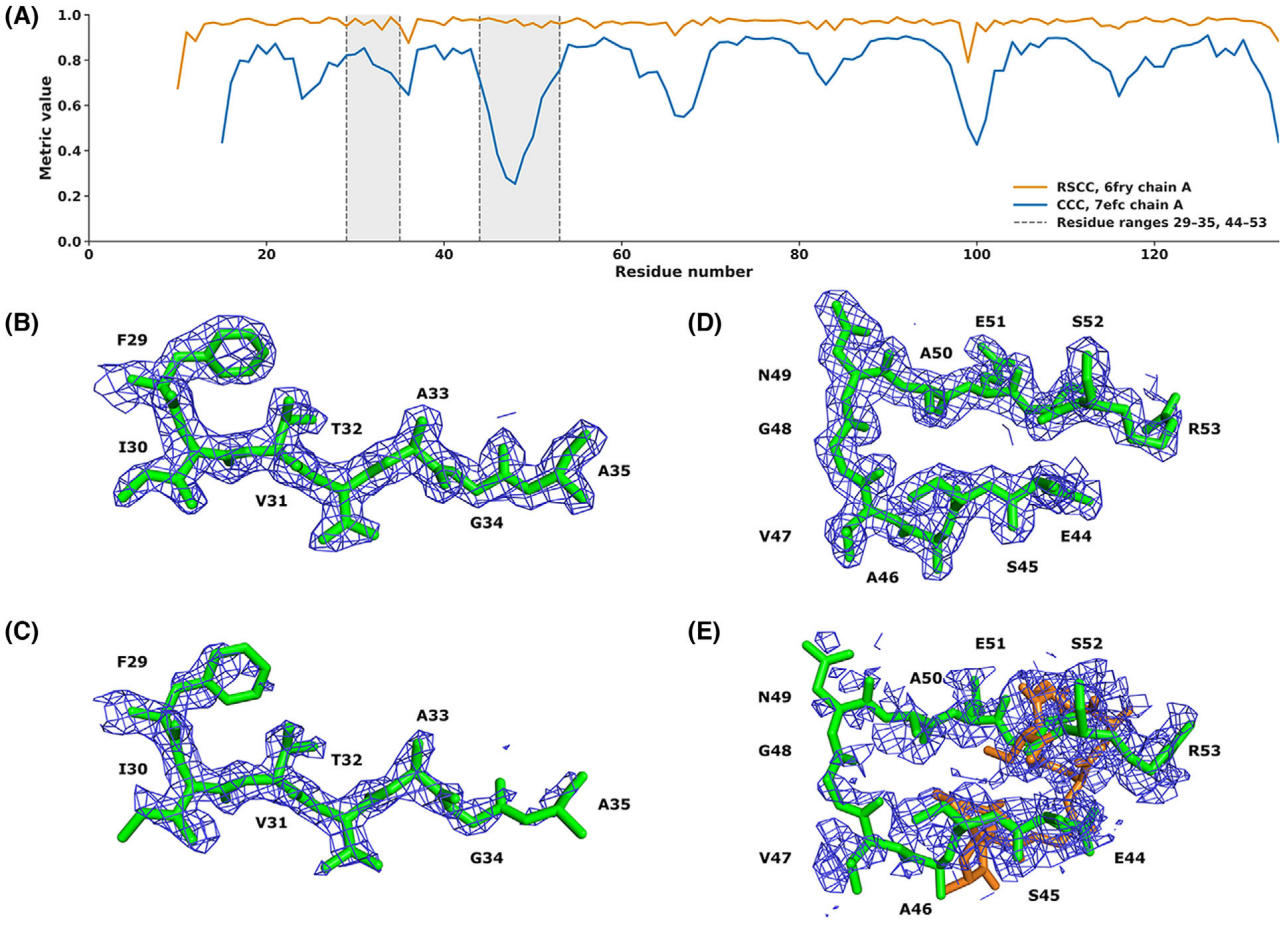
Comparison of local agreement between models and maps in two streptavidin structures: the X‐ray crystallographic model 6fry and the cryo‐EM model 7efc. (A) Per‐residue agreement between each model and its corresponding map, shown as the real‐space correlation coefficient (RSCC) for 6fry and the cross‐correlation coefficient (CC_mask_) for 7efc. RSCC values were obtained from the RCSB PDB Data API, and CC_mask_ values were obtained from the EMDB Validation Analysis API. Shaded areas correspond to the range displayed in the panels below. (B, D) 2mFo–DFc electron density map for 6fry contoured at 1σ, shown for residues 29–35 and 44–53, respectively. (C, E) Coulomb potential map EMD‐31083 for 7efc, shown for residues 29–35 and 44–53, respectively, contoured at 0.045, the author‐recommended level, in (C) and at 0.022 in (E). In (E), the alternative conformations of residues 45–52, modeled with occupancies of 0.5, are shown in orange. Panels B–E were prepared with the program pymex [32].

To compare the coordinates and the appearance of the electron density and EM Coulomb potential maps for structures determined at a comparable resolution, we selected the 1.7 Å crystal structure 6fry [31] with the lowest *R*_free_ (0.202) and the cryo‐EM structure 7efc. The newly developed program pymex [32] was used for this comparison. A comparison of the parameters describing model‐to‐map fit is shown in Fig. 4A. The fit is excellent for 6fry, whereas some areas of the EM map of 7efc fit the map less well. It should be stressed that these areas do not correspond to crystal contacts in 6fry. Selected map fragments are shown in Fig. 4B–E. Only a single protomer of each model is present in the PDB files and only a single orientation of each amino acid residue is present in the crystal structure. However, residues 45–52 in the cryo‐EM structure were modeled in two orientations, with 0.5 occupancy each (Fig. 4E). The crystal and cryo‐EM models can be superimposed with an RMSD of 0.38 Å for 120 Cα pairs (with orientation A of the dual‐conformation fragment in the cryo‐EM structure). The largest differences (~ 0.8 Å) are found for residues 82–83, neither of which is involved in intermolecular or crystal contacts in the crystal structure. Seventy‐two water molecules were modeled in the crystal structure, but only 39 are present in the cryo‐EM model. Twenty water molecules were placed in similar positions in the two models, within 0.5 Å of each other.

A direct comparison of crystal (7yqn) and cryo‐EM (7yqm) structures of malate synthase G, an 82 kDa enzyme, was published previously [25]. Its authors claimed that ‘the crystal and cryo‐EM structures are indistinguishable’ and that the quality of the cryo‐EM structure is ‘as high as that obtained from X‐ray crystallography’. That is indeed the case when geometric parameters and the fit to the maps are comparable. The crystal structure was determined at 1.6 Å resolution for a dimer in the asymmetric unit, with the RMSD for the 708 superimposed Cα pairs of the two subunits of 0.26 Å. The model is characterized by *R*_free_ of 0.172 and PQ1 of 0.95, with clashscore 1.28, sidechain outliers 0.26%, no Ramachandran outliers, and RSRZ 2.38%. Geometric quality parameters for the 2.9 Å cryo‐EM structure were comparable (clashscore 1.7, sidechain outliers 0.2%, no Ramachandran outliers). Fit between the model and Coulomb map was characterized by *Q*‐score of 0.548 and CC_mask_ of 0.78. Superposition of the cryo‐EM model and molecule A in the crystal structure resulted in RMSD of 0.45 Å for 711 Cα atom pairs. However, although the models are indeed similar, solvent was not modeled in the cryo‐EM structure, whereas as many as 1881 water molecules are present in the crystal structure.

### Modeling of solvent in crystal and cryo‐EM structures

**Fig. 5:**
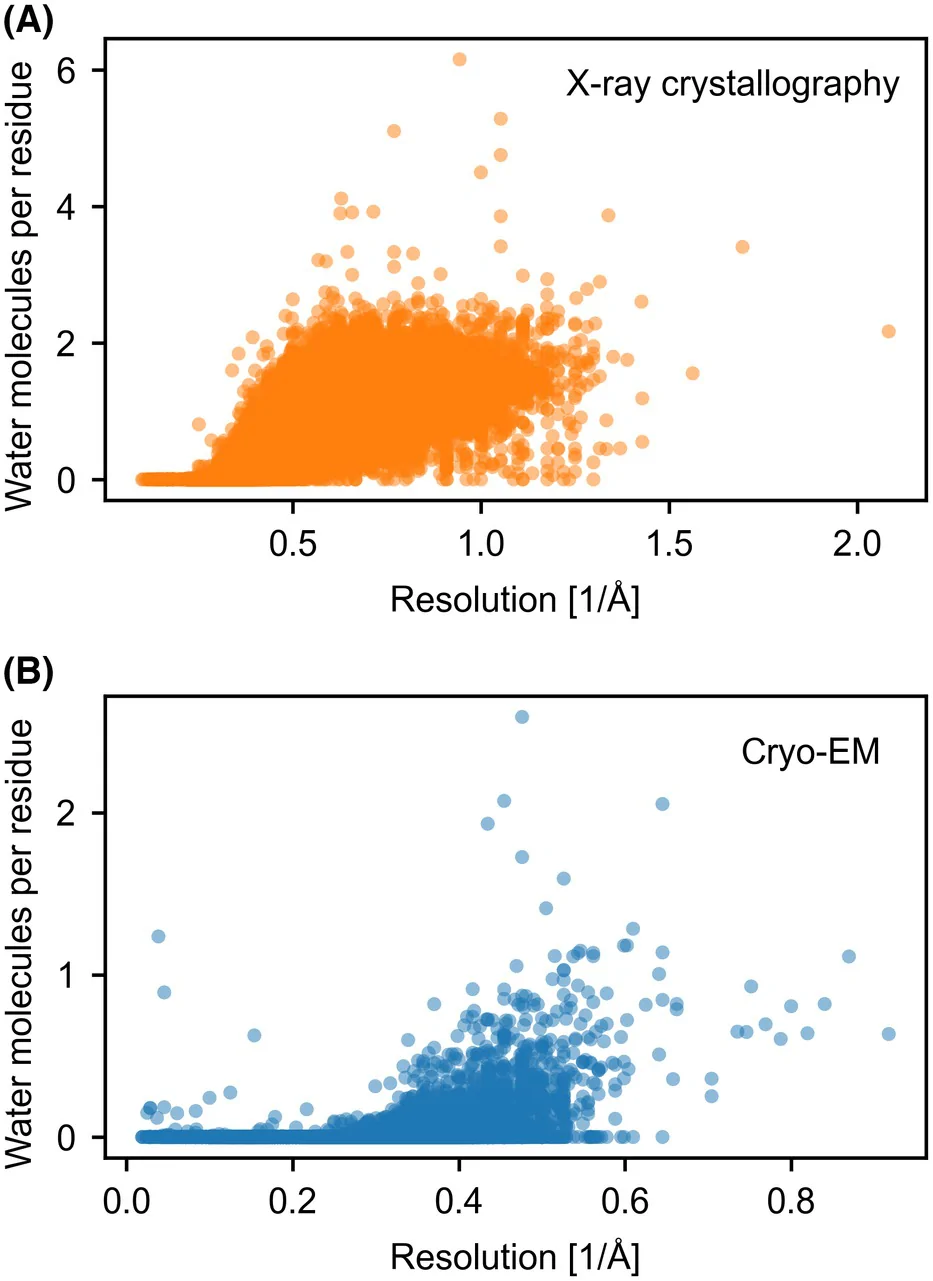
Ratios of the number of water molecules per modeled residues as a function of resolution of the crystal and cryo‐EM structures. (A) Crystal structures. (B) Cryo‐EM structures. Resolution is expressed as 1/*d*_min_ or FSC143 for crystal and cryo‐EM structures, respectively.

The point raised above brings up again a very important difference between PDB deposits based on crystal structures vs cryo‐EM. As mentioned earlier, only a small fraction of cryo‐EM deposits includes solvent, particularly water molecules. As many as 91.8% of crystal structures include water molecules (88.4% entries include 10 water molecules or more), but only 9.7% of cryo‐EM structures include any waters at all, with 6.8% including 10 water molecules or more. This is happening despite the well‐understood importance of the description of bound solvent for understanding of the mode of activity of the macromolecules under investigation. An excellent point illustrating the importance of solvent modeling was provided for acetylcholinesterase [33], for which no understanding of the function of the active site gorge could be gained without description of the location of over three dozen water molecules. However, as can be seen in Fig. 5, the fraction of waterless cryo‐EM structures is very high, although, as we previously pointed out, such structures are present in the PDB even for some crystal structures determined at high resolution [34]. It should also be noted that, despite its importance, solvent is not explicitly modeled by structure prediction programs such as AlphaFold2 or AlphaFold3. Unfortunately, many researchers outside the structural biology community do not first search the PDB for experimentally determined structures. Instead, they generate predicted models using structure prediction programs, even when suitable experimental structures are already available, thereby overlooking important information such as the presence of solvent molecules.

### Comparison of crystal and cryo‐EM structures of ribosomes

The evolution in the use of tools for structure determination of ribosomes represented a crucial transition from crystallography to cryo‐EM, thus meriting special attention for a comparison of the outcomes. Ribosomes are large macromolecular assemblies consisting of three or four RNA chains that contain some heavily modified nucleotide bases, as well as several dozen proteins. Functional ribosome is comprised of a small and a large subunit, which have been sometimes studied structurally in isolation, whereas a majority of the currently available structures represent complete ribosomal particles. Ribosome structures were first determined by X‐ray crystallography in the late 1990s [35, 36, 37], leading to the award of the 2009 Nobel Prize in Chemistry to Ada Yonath, Tom Steitz, and Venki Ramakrishnan. A total of 612 crystal structures of complete ribosomes or their individual subunits originating from different organisms, as well as of complexes with a variety of ligands, were deposited in the PDB between 2000 and 2025 (Table S3). The first medium‐resolution structure to be determined by cryo‐EM (PDB ID 3j6b) represented the large subunit of yeast mitochondrial ribosome, solved at the nominal resolution of 3.2 Å [38]. From that point on, cryo‐EM has become the preferred method for structural characterization of ribosomes, resulting in 1816 such deposits by the end of 2025 (Table S4). The reasons for this switch can be traced to difficulties in growing high‐quality crystals of ribosomes (see below), to the requirement for much less sample for cryo‐EM than for crystallography, and for the accelerated timeline of experiments that utilize cryo‐EM.

**Fig. 6:**
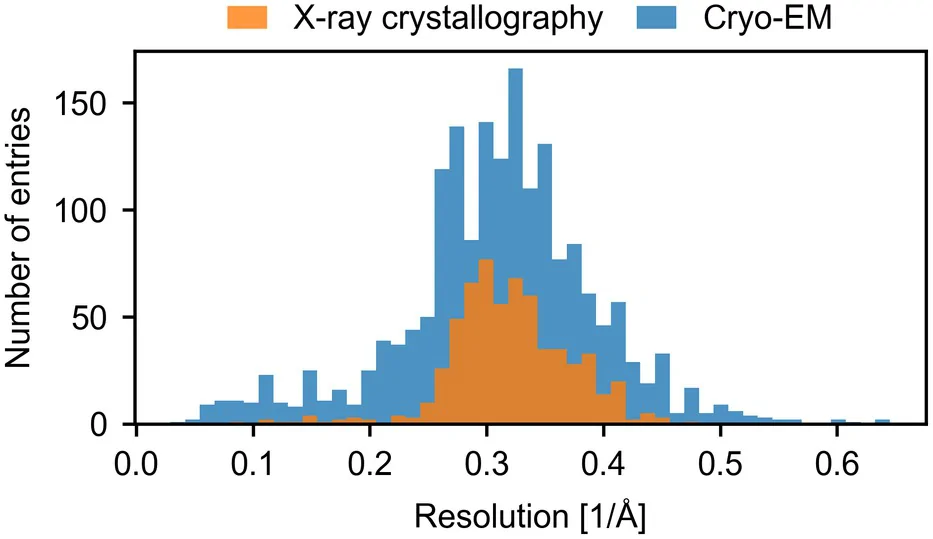
Distribution of resolution of the crystal and cryo‐EM structures of ribosomes, including complete ribosomes as well as individual small and large ribosomal subunits. Resolution is expressed as 1/*d*_min_ or FSC143 for crystal and cryo‐EM structures, respectively.

Resolution of the ribosome structures determined by either technique is quite varied (Fig. 6). The most typical resolution for either technique is ~ 3 Å, but the resolution range is much wider for structures obtained by electron microscopy (1.55–34 Å) than by crystallography (2.1–11.5 Å).

**Fig. 7:**
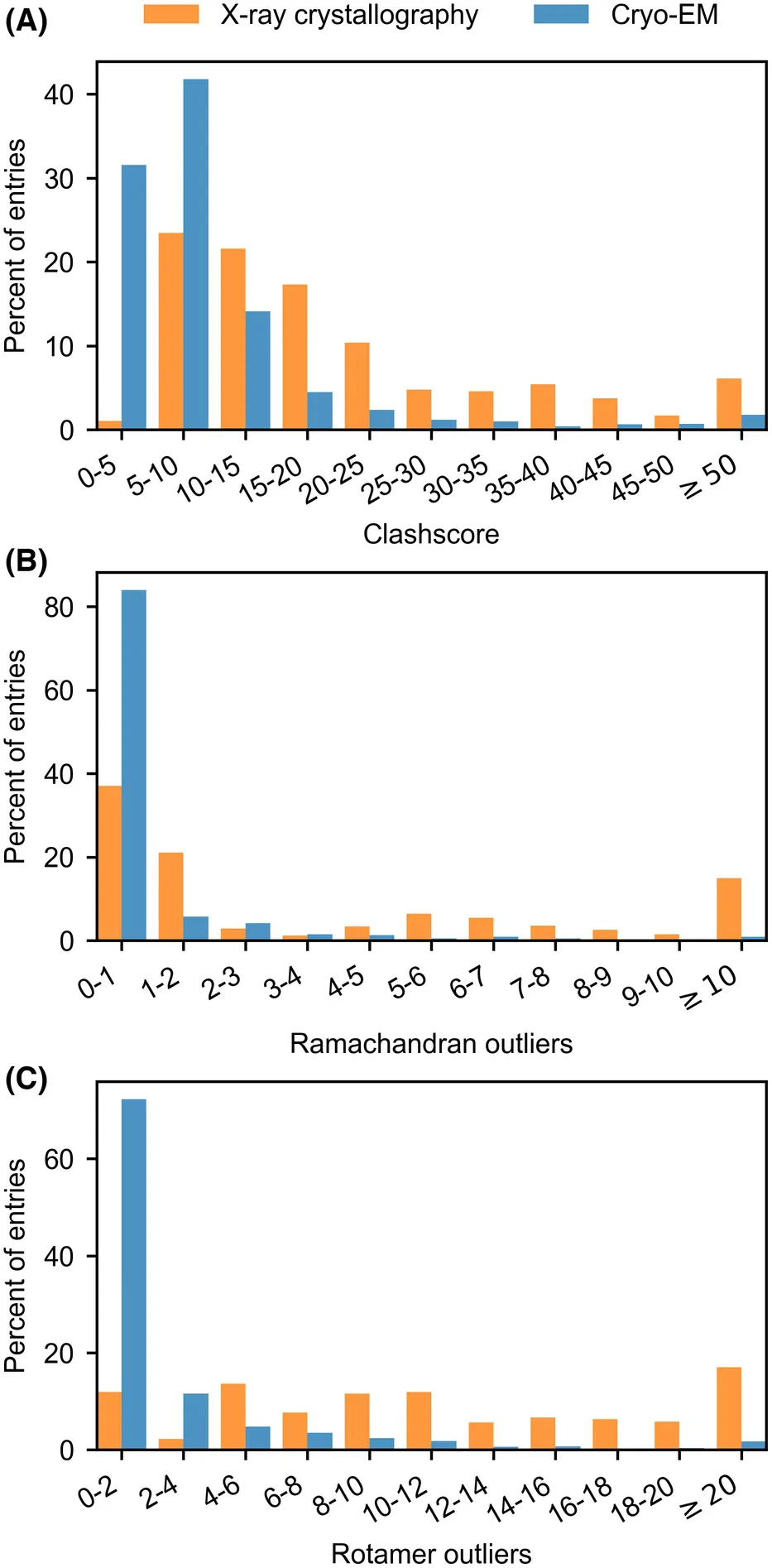
Histograms of the distribution of clashscore (A), Ramachandran (B), and rotamer (C) outliers (%), for X‐ray crystal and cryo‐EM structures of ribosomes determined between 2000 and 2025. The *y*‐axis is presented on a logarithmic scale.

Comparison of the quality of the crystal and cryo‐EM structures of ribosomes provides interesting data on the relative advantages of these two techniques as applied to well‐ordered large macromolecules. All geometric quality indicators are uniformly inferior for the crystal structures as opposed to the structures determined by cryo‐EM, with exceptions provided only by some structures determined at the highest resolution. This is true in particular for the Ramachandran and rotamer outliers, as well as for clashscore values (Fig. 7). The RNA backbone suite metric for the crystal structures ranges from the low of 0.24 (yeast 80S ribosome at 4.0 Å resolution; 4v7r) to the high of 0.69 (30S *T. thermophilus* ribosomal subunit at 4.5 Å resolution; 1i95). For cryo‐EM structures, this parameter ranged from 0 (2ftc, 2rdo, 4adx, 4v47, 4v48, 4v7f, and 5zzm; all these structures were at low resolution of 6.5–12.5 Å) to 0.69 [6wd7, 6wdh, and 6wdi; these are medium resolution (3.9–4.3 Å) structures].

**Fig. 8:**
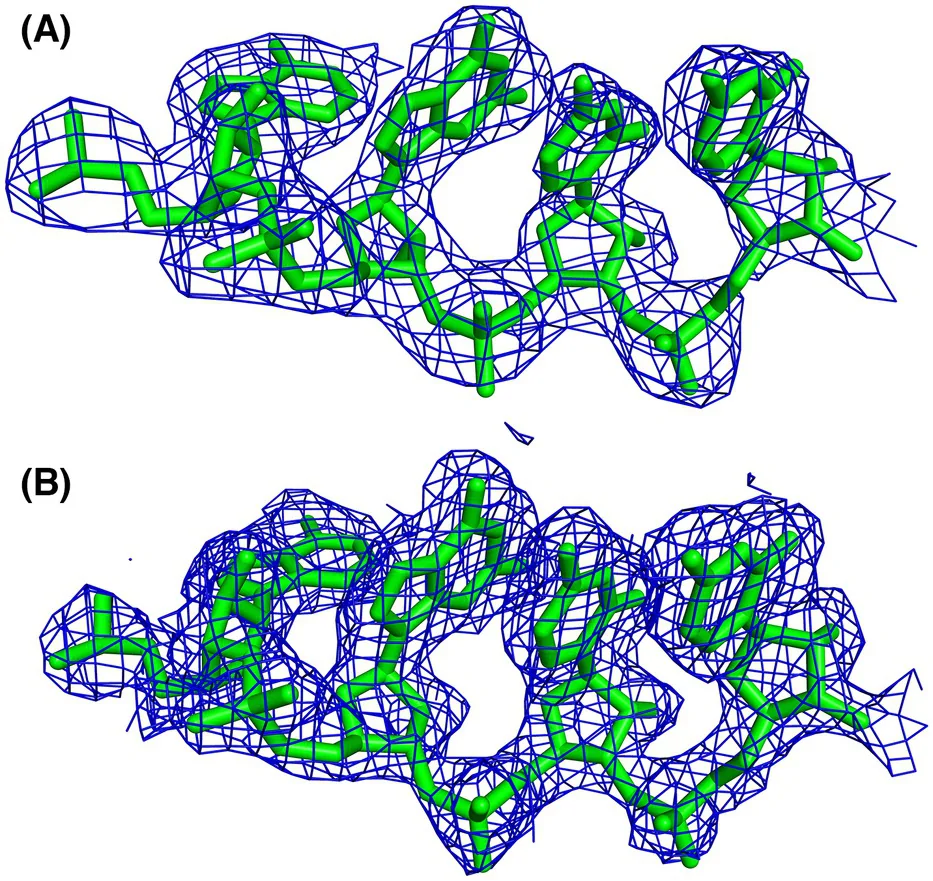
Comparison of local agreement between models and maps in the X‐ray crystallographic model 4ybb and the cryo‐EM model 7k00. Both panels show nucleotides 1067–1070, selected from chain AA in 4ybb and chain A in 7k00. (A) 2mFo–DFc electron density map for 4ybb contoured at 1σ. (B) Coulomb potential map EMD‐22586 for 7k00 contoured at the EMDB recommended level of 0.028. Figure was prepared with pymex.

For direct comparison of ribosome structures determined by cryo‐EM and by crystallography, we selected models of the 70S *E. coli* ribosome. Its crystal structure (4ybb) was determined at 2.1 Å resolution [39] and refined to *R*_free_ of 0.246 and PQ1 of 0.208. Individual values of the geometric and stereochemical parameters are all on the poor side, with clashscore 5, Ramachandran outliers 1.6%, sidechain outliers 7.1%, and RSRZ outliers 31.0%. However, the RNA backbone quality indicator of 0.55 suggests a reasonably good stereochemistry of the model. The crystal structure includes 7750 water molecules and 463 Mg^2+^ ions. The cryo‐EM structure 7k00, determined later by the same laboratory [40], was clearly of higher quality (0.1% Ramachandran outliers, 6.0% sidechain outliers, RNA backbone quality 0.58). Modeled solvent included 7248 water molecules and 309 Mg^2+^ ions. A comparison of selected regions of the electron density and EM maps is shown in Fig. 8. The maps, showing a fragment of the interior RNA chain, appear to be of comparable quality.

### How crystal and cryo‐EM structures of ribosomes were used to improve model quality

The currently available highest‐resolution (1.55 Å) cryo‐EM structure of *E. coli* ribosome (9q87; [41]) provides an excellent illustration of the progress in structure determination of these large molecular assemblies that was made possible thanks to the utilization of both crystallography and cryo‐EM, notching up a concomitant improvement in structure quality. The starting point of this series of structures was four crystal structures (3r8s, 3r8t, 4gd1, 4gd2) that were subsequently replaced by a single deposit 4v9d at 3 Å resolution [42]. The crystals contained two independent ribosomal particles in the asymmetric unit of space group *P*2_1_2_1_2_1_ with a total of 292 354 nonhydrogen atoms. The quality indicators of the structure 4v9d were not stellar, with clashscore of 42, 22% of sidechain outliers, and 2.2% RSRZ outliers. The same authors later redetermined this structure at 2.1 Å resolution (4ybb; [39]), but the quality parameters were still on the poorer side, in particular 31% of RSRZ outliers. According to the PDB, that model was subsequently rerefined at 2.9 Å resolution, however, without much improvement to the quality metrics (PDB ID 6i7v; [43]). We might question whether the connection between 4ybb and 6i7v is correct, since it is not mentioned in the publication linked to the latter model and the details of data collection are different for the two deposits.

The crystal structure 4ybb was subsequently used by the original authors for the determination of the cryo‐EM structure at 2 Å resolution (PDB ID 7k00; [40]). The geometry parameters of that structure were good, although the number of sidechain outliers (6.0%) was significant. The model was characterized by a *Q*‐score of 0.677 and CC_mask_ of 0.8282. The crystal structure 4ybb was also utilized later by another research group to assist in fitting the ribosome model to the cryo‐EM map at a nominal resolution of 1.55 Å (8b0x; [44]). This structure is much more idealized in terms of its geometry and the high quality of the cryo‐EM map even allowed proposing an unexpected G‐C base‐pair swap at positions G2210 and C2216 (MRE600 numbering) in helix 79 of the 23S rRNA. The value of *Q*‐score was 0.767 and of CC_mask_ 0.793. Despite the high quality of this structure, it was noticed that the identification of some solvent molecules and ions (1 Zn^2+^, 361 Mg^2+^, 168 K^+^, and 11 461 water molecules) was problematic [45]. That observation motivated another research group to rerefine the model using the publicly available data for 8b0x, mainly with the aim of providing the best possible description of the location of Mg^2+^ ions. The number of solvent molecules (almost doubled to 21 397) and metal cations (403 Mg^2+^, 231 K^+^) in the reinterpreted structure changed very significantly. Whereas the resolution of the resulting structure (9q87; [41]) remained the same, the quality indicators became significantly better. Interestingly, whereas CC_mask_ is the same as in the starting structure, the *Q*‐score is surprisingly lower, 0.740 vs 0.793. This is at present the highest‐resolution structure of any ribosome in the PDB.

## Discussion

Utilization of different structure determination methods has undergone major evolution during the last decade. In addition to cryo‐EM becoming the dominant experimental technique for studies of larger macromolecules and their assemblies, application of AI methods such as AlphaFold2 [3] has become routine, although the accuracy of predicted structures still cannot match that of their experimental counterparts and the resulting models usually do not include solvent [46]. As emphasized by data summarized in Fig. 2A, crystallography and cryo‐EM are routinely applied to targets differing in their sizes, although some exceptions to these rules can be found. Additional advantage of cryo‐EM is also the ability to observe simultaneously multiple conformations of the investigated molecules in the same experiment.

Although the steps in the initial determination of crystal and cryo‐EM structures are very different, refinement of the preliminary models utilizes the same software, such as Phenix [47] or REFMAC5 [48]. Since the refinement programs restrain stereochemical parameters to idealized targets, it is not surprising that a vast majority of both crystal and cryo‐EM structures do not exhibit large departures from the expected values. Crystal structures seem to exhibit more Ramachandran and rotamer outliers (Fig. 3), although they have lower clashscores, despite the fact that the latter are influenced by crystal contacts that do not affect structures determined by cryo‐EM. This trend does not apply to the special case of ribosome structures which are almost uniformly better when determined by cryo‐EM. This may be the effect of both the difficulties of growing high‐quality ribosome crystals, as well as of the fact that the crystal structures were to be determined first, using only medium‐resolution diffraction data that were available at that time. As shown by the examples discussed in the Comparison of crystal and cryo‐EM structures of ribosomes section, such improvement in structure quality happens when earlier results form the basis for subsequent structure determination, even by a different method.

A major difference between the models obtained by crystallography and cryo‐EM is the representation of solvent. Whereas over 90% of crystal structures include solvent, over 90% of cryo‐EM structures do not. This major difference is exacerbated by the much larger average size of the structures determined by cryo‐EM compared to structures determined by crystallography (Fig. 2A). Although the cryo‐EM ribosome structure 9q87 includes 21 397 water oxygen atoms among its 166 869 nonhydrogen atoms, over 250 cryo‐EM‐derived models (some also representing ribosomes) include only a single water molecule. Such an outcome is closely related to the resolution of the data used for structure solution, as has been recently discussed for crystal structures [34].

The trend illustrated in Fig. 1 suggests that macromolecular structure determination using cryo‐EM might be on track to eclipse the use of X‐ray crystallography. The direct ‘product’ of each technique is a ‘molecular map’, namely the electron density map from X‐ray diffraction and Coulomb potential map (which is nonzero even for the naked proton, H^+^) from cryo‐EM. Those experimental maps are then interpreted with a set of atomic coordinates to produce molecular models. As we have discussed here, each of these techniques has its own advantages and limitations that make them not quite interchangeable. Cryo‐EM is most applicable to the investigation of large macromolecules and their assemblies at medium resolution, whereas the strength of crystallography is still in illuminating the structural details of smaller macromolecules, with unsurpassed resolution and accuracy.

Important advantages of using cryo‐EM include independence from the uncertain nature of crystallization and the ability to separate different states of the macromolecules, captured in a single data set. However, structures at resolution lower than 3.2 Å (the median for cryo‐EM) are too crude for detailed description of intricate hydrogen bonds or for defining distances of nonbonded atoms with accuracy better than 0.1 Å. Since structures determined by both techniques rely currently very heavily on prior knowledge (via the use of previously published models for structure solution and utilization of idealized geometry in restrained refinement), it is not surprising that the geometrical and stereochemical parameters of structures deposited in the PDB in the last decade are usually quite acceptable (Fig. 3). However, for some specific systems, such as the ribosomes that provide a particular example, cryo‐EM is without doubt the method of choice for structure determination.

It comes as a positive surprise that the quality of the cryo‐EM structures is quite high. A speculative explanation of this observation might be that (a) the refinement methods are nowadays very powerful at yielding structures with high‐quality geometry and a good fit to the experimental maps; (b) since there is no real phase problem for cryo‐EM structures, better maps may inherently produce better structures; (c) overall, the cryo‐EM structures are actually more homogeneous than X‐ray crystal structures; this might be due to the cryo‐EM methodology, where the images are first segregated into homogenous 2D and 3D classes, thus treating structures ‘caught’ in slightly different conformations separately, with each of the classes consisting of conformationally very homogeneous set of molecules.

Although the field of X‐ray crystallography seemed (several times!) to have reached its apex, and even has been predicted (also several times) to be already dead, it keeps arising from the dead again and again. The current revolution has been brought about by new generation synchrotron beamlines, allowing for complete data sets to be collected in less than a second and promoting serial measurements (SSX) where it even does not matter if the serial samples are isolated single crystals, or crystallization slurries, or indeed even crystallization plates. Precise sample delivery methods also allow for time‐resolved studies, where kinetic processes taking place within protein crystals can be mapped in real time. But even this revolution is already overshadowed by another advancement that is possible at X‐ray free electron lasers (XFELs), where the beam of X‐ray photons is 12 orders of magnitude brighter than with the brightest synchrotrons, thus allowing for femtosecond exposure times and nanometer crystals, or indeed to reduce the sample size to the scale of a single macromolecule with sufficiently large mass.

By comparison, the field of high‐resolution single‐particle imaging cryo‐EM is at a very different stage of development. We can expect that there is still a lot of potential for the development of the very hardware of the microscope. With detectors already counting single electrons, it is not clear how much improvement will be achieved in reducing such negative effects as sample damage. Another problem is sample vibration. Both effects could be reduced by less intense electron beams. And there is of course huge potential for the development of better software, with no doubt that the algorithms will benefit from AI assistance. And if quantum computing becomes a reality, time‐consuming computations would be substantially reduced.

The above prophecies may not be realized immediately (if at all!). But one thing is certain: both X‐ray crystallography and cryo‐EM are still in very good shape and will continue to be crucial tools of structural biology for quite some time, with the center of gravity shifting toward cryo‐EM. The availability of both techniques has clearly resulted in major advances during the last decade and bodes very well for the future.

## Conflict of interest

WM declares that he has had involvement in the development of software and data management and data‐mining tools; some of these have been commercialized by HKL Research. W Minor is the cofounder of HKL Research and a member of the board. The authors have no other relevant affiliations or financial involvement with any organization or entity with a financial interest in or financial conflict with the subject matter or materials discussed in the manuscript apart from those disclosed.

## Author contributions

AW, WM, and MJ designed the project. All authors analyzed and interpreted the data and wrote the manuscript.

## Supporting information


**Table S1.** Crystal structures released by the PDB between 1 January 2015 and 31 December 2025.

**Table S2.** Cryo‐EM structures released by the PDB between 1 January 2015 and 31 December 2025.

**Table S3.** Crystal structures in the PDB of complete ribosomes from different organisms and organelles or at least their small or large subunits, but excluding isolated ribosomal proteins.

**Table S4.** Cryo‐EM structures in the PDB of complete ribosomes from different organisms and organelles or at least their small or large subunits, but excluding isolated ribosomal proteins.

## Data Availability

All data referred to in this manuscript is contained in the Supplementary Tables or can be downloaded directly from the Protein Data Bank.
